# Serum amino acid concentrations and clinical outcomes in smokers: SPIROMICS metabolomics study

**DOI:** 10.1038/s41598-019-47761-w

**Published:** 2019-08-06

**Authors:** Wassim W. Labaki, Tian Gu, Susan Murray, Jeffrey L. Curtis, Larisa Yeomans, Russell P. Bowler, R. Graham Barr, Alejandro P. Comellas, Nadia N. Hansel, Christopher B. Cooper, Igor Barjaktarevic, Richard E. Kanner, Robert Paine, Merry-Lynn N. McDonald, Jerry A. Krishnan, Stephen P. Peters, Prescott G. Woodruff, Wanda K. O’Neal, Wenqi Diao, Bei He, Fernando J. Martinez, Theodore J. Standiford, Kathleen A. Stringer, MeiLan K. Han

**Affiliations:** 1 Division of Pulmonary and Critical Care Medicine, University of Michigan, Ann Arbor, MI USA; 2 Department of Biostatistics, University of Michigan, Ann Arbor, MI USA; 3 Medical Service, VA Ann Arbor Healthcare System, Ann Arbor, MI USA; 4 Biochemical NMR Core and the NMR Metabolomics Laboratory, College of Pharmacy, University of Michigan, Ann Arbor, MI USA; 5 Division of Pulmonary, Critical Care and Sleep Medicine, National Jewish Health, Denver, CO USA; 6 Division of Pulmonary, Allergy and Critical Care Medicine, Columbia University, New York, NY USA; 7 Division of Pulmonary, Critical Care and Occupational Medicine, University of Iowa, Iowa City, IA USA; 8 Division of Pulmonary and Critical Care Medicine, Johns Hopkins University, Baltimore, MD USA; 9 Department of Medicine, University of California Los Angeles, Los Angeles, CA USA; 10 Division of Pulmonary Medicine, University of Utah, Salt Lake City, UT USA; 11 Division of Pulmonary, Allergy and Critical Care Medicine, University of Alabama at Birmingham, Birmingham, AL USA; 12 Department of Genetics, University of Alabama at Birmingham, Birmingham, AL USA; 13 Division of Pulmonary, Critical Care, Sleep and Allergy, University of Illinois at Chicago, Chicago, IL USA; 14 Section on Pulmonary, Critical Care, Allergy and Immunologic Diseases, Wake Forest University, Winston-Salem, NC USA; 15 Division of Pulmonary, Critical Care, Allergy and Sleep Medicine, University of California San Francisco, San Francisco, CA USA; 16 Marsico Lung Institute, Department of Medicine, University of North Carolina at Chapel Hill, Chapel Hill, NC USA; 17 Department of Respiratory Medicine, Peking University Third Hospital, Beijing, China; 18 Division of Pulmonary and Critical Care Medicine, Weill Cornell Medical College, New York, NY USA; 19 Department of Clinical Pharmacy, College of Pharmacy, University of Michigan, Ann Arbor, MI USA

**Keywords:** Chronic obstructive pulmonary disease, Predictive markers, Translational research

## Abstract

Metabolomics is an emerging science that can inform pathogenic mechanisms behind clinical phenotypes in COPD. We aimed to understand disturbances in the serum metabolome associated with respiratory outcomes in ever-smokers from the SPIROMICS cohort. We measured 27 serum metabolites, mostly amino acids, by ^1^H-nuclear magnetic resonance spectroscopy in 157 white ever-smokers with and without COPD. We tested the association between log-transformed metabolite concentrations and one-year incidence of respiratory exacerbations after adjusting for age, sex, current smoking, body mass index, diabetes, inhaled or oral corticosteroid use, study site and clinical predictors of exacerbations, including FEV_1_% predicted and history of exacerbations. The mean age of participants was 53.7 years and 58% had COPD. Lower concentrations of serum amino acids were independently associated with 1-year incidence of respiratory exacerbations, including tryptophan (β = −4.1, 95% CI [−7.0; −1.1], p = 0.007) and the branched-chain amino acids (leucine: β = −6.0, 95% CI [−9.5; −2.4], p = 0.001; isoleucine: β = −5.2, 95% CI [−8.6; −1.8], p = 0.003; valine: β = −4.1, 95% CI [−6.9; −1.4], p = 0.003). Tryptophan concentration was inversely associated with the blood neutrophil-to-lymphocyte ratio (p = 0.03) and the BODE index (p = 0.03). Reduced serum amino acid concentrations in ever-smokers with and without COPD are associated with an increased incidence of respiratory exacerbations.

## Introduction

Chronic obstructive pulmonary disease (COPD) is a prevalent condition typically caused by exposure to smoking or other noxious particles and characterized by chronic respiratory symptoms and irreversible airflow obstruction^1^. COPD is a highly heterogeneous disorder; some patients experience frequent respiratory exacerbations while other do not^2,3^, not all patients progress towards severe emphysema regardless of the severity of airflow obstruction, and women tend to have more rapid disease progression than men^4^. This complex heterogeneity cannot be fully captured by lung function measurement alone or by any single biomarker.

Metabolomics is an emerging science that involves the simultaneous measurement of endogenous low molecular weight compounds (≤1,500 Daltons) in a biological specimen^5,6^. Not only does the metabolome interact with and reflect the activity of the genome, epigenome and proteome, but it is also influenced by one’s diet, lifestyle and medications^5^. Therefore, given its capture of gene function, enzyme activity and physiologic changes, the study of metabolomics has the potential to inform the clinical heterogeneity of COPD phenotypes. While human studies conducted to date have showed that metabolomics analysis of the serum, plasma, urine and exhaled breath condensate can discriminate between never smokers, smokers with COPD and smokers without COPD^7–11^, the relationship between metabolic profiles and the clinical heterogeneity of COPD has not yet been fully characterized.

A key outcome in the natural history of COPD is respiratory exacerbations which are associated with accelerated lung function decline^12^ and increased mortality^13^. It has been established that plasma metabolomics become significantly disturbed during acute exacerbation events^14^. We hypothesized that an altered serum metabolome among ever-smokers in the stable state would be associated with an increased risk of future respiratory exacerbations. To test this hypothesis, we conducted a prospective analysis of the relationship between the baseline serum metabolome measured by ^1^H-nuclear magnetic resonance (NMR) spectroscopy and the incidence of exacerbations over one year of follow-up in participants of the Subpopulations and Intermediate Outcome Measures in COPD Study (SPIROMICS) cohort^15^. We also explored cross-sectional associations between serum metabolomics and other pulmonary parameters such as lung function, extent of emphysema on chest computed tomography (CT) and exertional capacity.

## Results

### Description of participants and metabolites

The 157 participants included in this analysis had a mean age of 53.7 years, with 56.8% being current smokers and 58.0% having COPD confirmed on spirometry (Table 1). The prevalence of diabetes mellitus in our cohort was 7.1%, and 30.5% of participants were on inhaled corticosteroids. Among the 138 participants for whom information on prospective respiratory exacerbations was available, 109 reported no exacerbations, while 29 experienced at least one exacerbation over one year of follow-up. Participants in the exacerbation group were more likely to have COPD (especially with spirometry grade Global Initiative for Chronic Obstructive Lung Disease (GOLD) 3–4 severity), more emphysema on chest CT and diabetes mellitus, and were more likely to be using an inhaled corticosteroid (Table 1). NMR spectroscopy reliably identified and quantified 27 serum metabolites in all participants. Measured metabolites were mostly amino acids or amino acid derivatives, but also included glucose, choline and three organic acids (2-hydroxybutyrate, citrate and lactate).

**Table 1:** Baseline characteristics of participants.

	All participants (n = 157)*	No exacerbation (n = 109)	≥1 exacerbation (n = 29)	p-value**
**Demographics**				
Age	53.7 (4.8)	53.7 (4.8)	54.4 (4.5)	0.50
Female	79 (50.3%)	52 (47.7%)	18 (62.1%)	0.17
**Smoking history**				
Smoking pack-years	45.8 (35.1)	45.2 (39.5)	50.3 (25.6)	0.40
Current smoking	88 (56.8%)	59 (55.1%)	17 (58.6%)	0.74
**Pulmonary parameters**				
FEV_1_/FVC < 0.7	91 (58.0%)	58 (53.2%)	26 (89.7%)	0.0004
GOLD spirometry grade 1–2	62 (39.5%)	45 (41.3%)	14 (48.3%)	0.50
GOLD spirometry grade 3–4	29 (18.5%)	13 (11.9%)	12 (41.4%)	0.0003
6MWD (m)	445.0 (138.5)	460.6 (136.1)	403.1 (153.1)	0.051
% emphysema on CT	5.2 (9.0)	3.8 (6.7)	9.2 (12.7)	0.04
**Exacerbation predictors**				
FEV_1_% predicted	78.7 (27.4)	83.1 (24.9)	57.8 (23.2)	<0.0001
≥1 exacerbation during previous year	30 (19.1%)	16 (14.7%)	14 (48.3%)	0.0001
SGRQ score	35.0 (22.2)	30.0 (20.4)	57.3 (13.1)	<0.0001
WBC count (thousands)	7.7 (2.5)	7.6 (2.4)	8.6 (3.0)	0.07
GERD	55 (35.0%)	39 (35.8%)	16 (55.2%)	0.07
**Medication use**				
Inhaled corticosteroids	47 (30.5%)	25 (23.4%)	18 (62.1%)	<0.0001
Oral corticosteroids	2 (1.3%)	1 (0.9%)	1 (3.4%)	0.32
**Metabolic parameters**				
BMI (kg/m^2^)	27.7 (4.9)	27.9 (5.0)	28.1 (4.6)	0.79
Diabetes mellitus	11 (7.1%)	5 (4.7%)	5 (17.2%)	0.02

Data are expressed as mean (SD) for continuous variables and count (percentage) for categorical ones. FEV_1_, forced expiratory volume in the first second; FVC, forced vital capacity; GOLD, Global Initiative for Chronic Obstructive Lung Disease; 6MWD, 6-minute walking distance; CT, computed tomography; SGRQ, St. George’s Respiratory Questionnaire; WBC, white blood cell; GERD, gastroesophageal reflux disease; BMI, body mass index.

*Exacerbation data missing in 19 participants.

**p-value comparing characteristics between group with no exacerbation and group with ≥1 exacerbation.

### Association of serum metabolites with incident respiratory exacerbations

**Figure 1:**
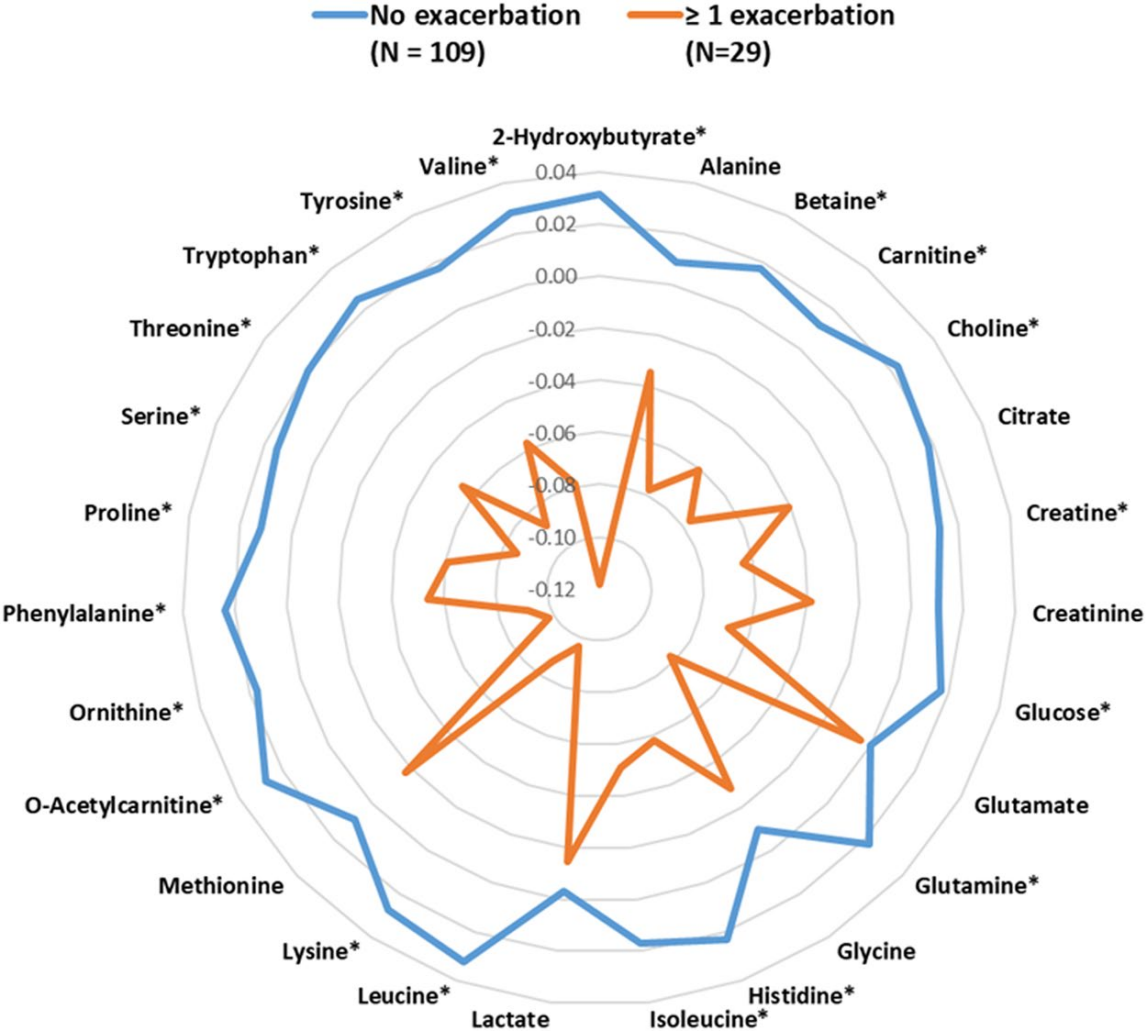
Radar plot showing mean-centered and range-scaled log-transformed metabolite concentrations by exacerbation group. Asterisks (*) denote statistical significance at the 10% false discovery rate.

**Figure 2:**
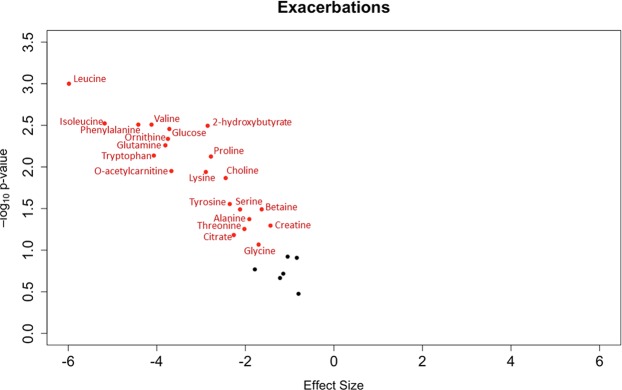
Volcano plot showing the adjusted association between log-transformed metabolite concentrations and incident respiratory exacerbations. Red dots represent metabolites that are statistically significant at the 10% false discovery rate. Black dots represent metabolites that are not statistically significant.

Compared to participants without exacerbations, those with at least one exacerbation had significantly lower serum concentrations for 20 of the 27 identified metabolites (Fig. 1 and Supplementary Table S1). This negative association between baseline metabolite concentrations and incident respiratory exacerbations persisted in adjusted analyses (Fig. 2) where a total of 21 metabolites were statistically significant after correction for multiple comparisons, including tryptophan (β = −4.1, 95% CI [−7.0; −1.1], p = 0.007) and the branched-chain amino acids (leucine: β = −6.0, 95% CI [−9.5; −2.4], p = 0.001; isoleucine: β = −5.2, 95% CI [−8.6; −1.8], p = 0.003; and valine: β = −4.1, 95% CI [−6.9; −1.4], p = 0.003). Multiple imputation results for missing concentrations of metabolites yielded similar results except for histidine which reached statistical significance in this sensitivity analysis (Supplementary Fig. S2).

### Association of serum metabolites with pulmonary parameters

**Figure 3:**
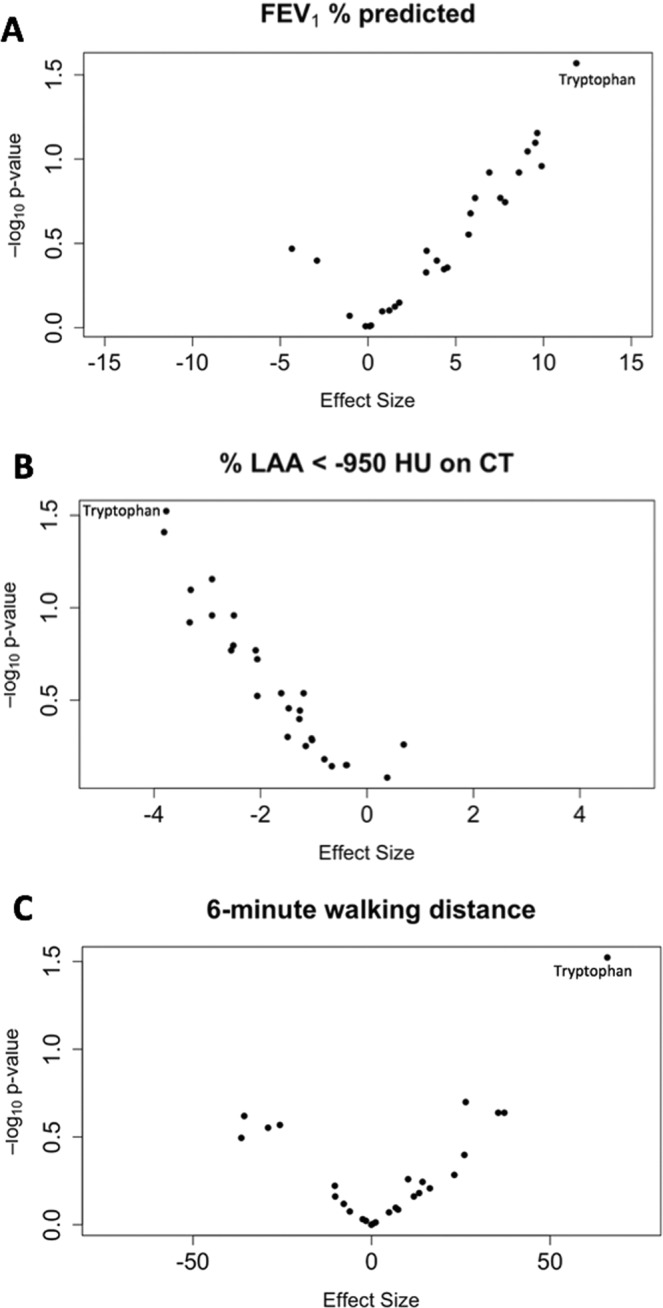
Volcano plots showing the association between log-transformed metabolite concentrations and FEV1% predicted (**A**), % low attenuation area (LAA) <−950 Hounsfield Units (HU) on chest computed tomography (CT) (**B**), and 6-minute walking distance (**C**). Black dots represent individual metabolites.

In multivariable models, none of the metabolites were significantly associated with FEV_1_% predicted, % emphysema on chest CT or 6-minute walking distance (6MWD) after correction for multiple comparisons. Most serum metabolites were positively associated with the forced expiratory volume during the first second (FEV_1_) % predicted (Fig. 3A) and negatively associated with % emphysema (Fig. 3B). No trend was found when 6MWD was the outcome of interest (Fig. 3C). Notably, tryptophan had considerable associations with all pulmonary parameters: FEV_1_% predicted (β = 11.87; 95% CI [1.38, 22.36]; p = 0.03), % emphysema (β = −3.77; 95% CI [−7.17, −0.36]; p = 0.03) and 6MWD (β = 66.02; 95% CI [6.59, 125.45]; p = 0.03).

### Association of tryptophan with inflammatory and prognostic indices

Because tryptophan was consistently related to all outcomes, we tested its association with both blood neutrophil-to-lymphocyte ratio (NLR) and the Body mass index, airflow Obstruction, Dyspnea, Exercise capacity (BODE) index. After adjusting for relevant clinical covariates, tryptophan was inversely associated with NLR (β = −0.02; 95% CI [−0.03, −0.001]; p = 0.03) in all participants and with BODE (β = −0.04; 95% CI [−0.08, −0.003]; p = 0.03) in those with COPD.

## Discussion

In this NMR metabolomics analysis of current and former smokers in the SPIROMICS cohort, we found that reduced serum concentrations of amino acids are associated with a higher incidence of respiratory exacerbations. In addition, we show consistent and substantial associations between decreased tryptophan concentration and worse lung function, % emphysema on chest CT and exertional capacity. These preliminary findings support the concept of COPD as a systemic disorder and offer new insights into potential pathogenic pathways.

Circulating amino acids are involved in multiple physiologic functions, including protein synthesis, gluconeogenesis, immunity, and cell signaling^16^. Prior studies have described decreased serum concentrations of many amino acids in individuals with COPD compared to healthy subjects^17,18^. Our study further extends this knowledge by showing that lower amino acid concentrations are independently associated with worse clinical outcomes in ever-smokers. An untargeted liquid chromatography mass spectrometry (LC-MS)-based plasma metabolomics study of smokers from the COPDGene cohort also showed an inverse association between conccentrations of some amino acids (including tryptophan, tyrosine and glutamine) and exacerbation frequency^19^. This reduction in amino acids could be due to a consumptive process at the level of either or both the immune and musculoskeletal systems that rely on amino acids for their function, as described below. We cannot exclude a contribution from altered uptake of dietary sources as gut-resident bacteria can limit availability of amino acids, especially tryptophan^20^. However, given the tight regulation of levels of most serum amino acids in health, we consider this alternative explanation less likely, though meriting investigation.

The essential amino acid tryptophan regulates physiologic processes within multiple organs and systems. The amount of tryptophan that contributes to protein synthesis or to serotonin production is minimal as more than 95% is converted into kynurenine and related molecules, initially by indoleamine 2,3-dioxygenase (IDO), the rate-limiting extra-hepatic enzyme in the kynurenine pathway^20^. Kynurenine can reduce the activity of natural killer cells, antigen-presenting cells, and T cells. Conversely, immune cells themselves produce enzymes regulating the kynurenine pathway in response to pro-inflammatory cytokines and pathogens^20,21^. Gulcev and colleagues found decreased plasma tryptophan levels and increased IDO activity in COPD patients experiencing an acute exacerbation compared to COPD controls in the stable state^14^. One possible explanation of our results is that increased serum kynurenine dampens immune defenses to respiratory pathogens, predisposing to subsequent exacerbation events. Furthermore, tryptophan metabolites are major endogenous ligands of the aryl hydrocarbon receptor (AhR), a transcription factor with important immunomodulatory effects in the respiratory tract that is expressed in lung epithelial cells and a variety of immune cells^22^. Support for a generalized inflammatory milieu contributing to higher exacerbation risk in susceptible hosts comes from our finding of an inverse association between tryptophan and blood NLR, a well-studied predictor of COPD progression and outcomes^23^. Because all amino acids, not only tryptophan, are necessary for successful immune responses^24^, it is noteworthy that we found significant associations between concentrations of many of them and incident respiratory exacerbations, even after adjusting for relevant clinical covariates, including prior exacerbation history which is one of the strongest predictors of future exacerbations^25^.

Beyond their role in the immune system, amino acids are also major determinants of muscle mass, implying that body composition might mediate the association between decreased amino acid concentrations and poor pulmonary outcomes. Muscle dysfunction is prevalent in COPD and lower muscle mass is correlated with increased exacerbation risk as well as worse lung function, respiratory symptoms, exertional capacity and emphysema^26^. The branched-chain amino acids (leucine, isoleucine, valine) are particularly important contributors to muscle health, as they modulate oxidative use of glucose by muscles^27^. Serum levels of branched-chain amino acids have consistently been found to be lower in individuals with COPD^17,28,29^. Notably, one study showed increased quadriceps-to-plasma leucine content in COPD subjects compared to controls, suggesting increased muscle uptake as part of a consumptive process^30^. None of the metabolites identified in our study had a significant association with 6MWD; it is unclear whether the high baseline exertional capacity (mean 6MWD 445 meters) of our participants attenuated a potential association. In a small randomized placebo-controlled trial of COPD patients on long-term oxygen therapy, dietary supplementation with creatine and CoQ10 resulted in decreased dyspnea and improved functional performance^11^. Although amino acid supplementation holds promise, it still needs to be validated on a broad scale.

We found an inverse relationship between concentrations of most metabolites and severity of emphysema, although statistical significance was not met. Our results complement a report showing decreased tryptophan levels in COPD patients with predominantly emphysema versus those with predominantly airways disease^17^. Importantly, whether tryptophan or other amino acids mechanistically contribute to emphysema progression or lung function decline, remains unknown. Nonetheless, we showed a significant association between tryptophan concentrations and the BODE index, a validated and widely used predictor of mortality in individuals with COPD^31^.

Beyond identification of the serum amino acid metabolome as a biomarker of worse clinical parameters and outcomes in COPD, this metabolomics analysis provides important preliminary data to study some of the mechanisms involved in COPD pathogenesis. A molecular understanding of the disturbances affecting the metabolome may lead to a better understanding of COPD as a systemic condition. This is because of prevalent altered metabolic and body composition parameters in COPD, including cachexia, sarcopenia and osteoporosis in patients with emphysema, and overweight/obesity, diabetes mellitus and the metabolic syndrome in patients with chronic bronchitis. Given the complex heterogeneity of COPD, it is possible that different metabolic pathways are associated with specific clinical phenotypes^19^. Therefore, a comprehensive approach involving mechanistic studies, pathway analyses and integration of metabolomics data with data from other -omics sciences has the potential to identify specific pathogenic metabolic pathways. Once such pathways are identified, their individual components (e.g. gene expression, protein/enzyme activity, metabolite concentration) can be the subject of further studies to determine the etiology behind the observed alterations. This knowledge may lead to new therapeutic targets which are currently sorely needed in COPD.

Our study has a number of strengths, including its well-characterized and relatively large number of participants as well as the prospective collection of exacerbation events. We also acknowledge several limitations. Only white participants 40–60 years of age were included and less than 20% had a GOLD spirometry grade of ≥3. Therefore, our results are not generalizable to all smokers, including those with severe emphysema who may be cachexic and have substantially altered metabolomes. We could not assess the effects of diet and body composition beyond the body mass index (BMI) on the concentrations of serum metabolites, as data from food questionnaires and measurements of muscle mass and fat-free body mass were not available for our participants. This is particularly important because muscle mass could be a confounder underlying our findings, i.e. low muscle mass may lead to lower metabolite concentrations and also increase the risk of exacerbations. Furthermore, while we adjusted our models for the presence of diabetes mellitus, we did not have data available on the severity of diabetes and the use of insulin or oral hypoglycemic agents, which are factors that can affect systemic metabolism and therefore influence the association between metabolomics and exacerbation incidence. The use of NMR mostly detected amino acids and their derivatives; other classes of metabolites, especially lipids, could not be evaluated. In addition, 3-hydroxybutyrate and acetate were not included in this analysis due to aberrantly high concentrations in a subset of participants. Finally, we measured metabolites at a single point in time, precluding assessment of the association between temporal changes in the metabolome and longitudinal outcomes.

In summary, we showed that a reduction in serum amino acid concentrations is independently associated with an increased risk of respiratory exacerbations in ever-smokers with and without COPD. This association may be related to underlying systemic inflammation, immune dysregulation and/or skeletal muscle dysfunction. Further studies are needed to confirm our preliminary findings and tie them to results from other –omics sciences to better understand the pathogenic pathways involved.

## Methods

### Study participants and phenotypic measurements

The design of SPIROMICS, a multicenter longitudinal study funded by the National Health Lung and Blood Institute (ClinicalTrials.gov identifier: NCT01969344) has been previously reported^15^. In this metabolomics sub-study, participants were selected from 12 different sites to approximately reflect the entire cohort in terms of distribution of sex (equal numbers of males and females) and spirometry severity. To limit the effect of demographic heterogeneity on metabolomics results^32,33^, only white, ever-smoker (≥20 pack-years) participants, 40–60 years of age, were selected for this pilot analysis. Airflow obstruction was defined as a post-bronchodilator ratio of the forced expiratory volume in the first second to the forced vital capacity (FEV_1_/FVC) < 0.7. Spirometry tracings were reviewed to confirm adherence to American Thoracic Society and European Respiratory Society standards^34^. The severity grade of airflow limitation was determined in participants with COPD based on GOLD guidelines^1^. History of respiratory exacerbations in the year before study enrollment, smoking history, self-reported comorbidities, and medication use were all obtained from participants at the baseline visit. In addition, a 6-minute walk test and a high-resolution CT of the chest were acquired during that visit. Chest CTs were performed and analyzed according to the SPIROMICS quantitative CT lung assessment system protocol^35^. Extent of emphysema on CT was measured as the percent of lung volume with attenuation <−950 Hounsfield Units (HU) at full inspiration^36^. Prospective data on exacerbations was captured every three months through a structured telephone questionnaire. Exacerbations were defined as symptomatic respiratory events that necessitated health care utilization (clinic visit, Emergency Department visit or hospitalization) and treatment with antibiotics, systemic corticosteroids, or both. This definition was applied to both smokers with and without COPD as the latter group can experience significant morbidity from exacerbation-like events^37,38^.

### Serum sample processing and quantitative ^1^H-NMR metabolomics

Blood was collected from fasting participants by direct venipuncture, into no-additive red top tubes (BD Vacutainer®, Franklin Lakes, NJ, USA) in accordance with the SPIROMICS Manual of Operations. Blood was allowed to coagulate at room temperature for 30 minutes, after which it was centrifuged (3000 × g for 15 minutes). The resulting serum was aliquoted and stored at −80 °C. At the time of assay, samples were randomly assigned to a total of 14 batches and were thawed in an ice water bath. Pre-chilled methanol was added to each sample in a 2:1 ratio to remove proteins and macromolecules by precipitation. The aqueous fraction was lyophilized, suspended in deuterated water (D_2_O) and filtered (3KDa Pall Nanosep omega filters Sigma-Aldrich, St. Louis, MO, USA) to remove residual macromolecules, after which a known amount of formate was added as an internal standard as previously described^39,40^. On the day of NMR acquisitions, the pH was adjusted to a 6.5–7.5 range, and samples were transferred to NMR tubes. A one-dimensional ^1^H-NMR spectrum was generated for each sample using a 500 MHz spectrometer (Agilent Inc., Santa Clara, CA, USA) equipped with a 5-mm Agilent “One-probe” at the University of Michigan NMR Biochemical Core Laboratory. Spectra were acquired by 32 scans of the first increment of a ^1^H, ^1^H-NOESY pulse sequence^41^ and were analyzed using Chenomx NMR Suite 8.3 (Chenomx Inc., Edmonton, Alberta, Canada). We identified metabolites using the compound library of the Chenomx software and determined their concentrations relative to the internal standard. A member of the NMR Laboratory cross-checked the quality of each NMR spectrum, and 157 subjects with interpretable NMR spectra were included in this analysis (Supplementary Fig. S3).

### Statistical analysis

We reported baseline characteristics of participants as mean and standard deviation for continuous variables and as count and percentage for categorical ones. We assessed differences in characteristics between participants who had at least one respiratory exacerbation and those who did not experience any exacerbations over one year of follow-up using the two-sample t-test for continuous variables and the Chi-square test for categorical ones. We used a two-sample t-test to assess differences in metabolite concentrations between the two exacerbation groups. Results of this analysis were displayed on a radar plot by exacerbation group after the log-transformed concentrations of all metabolites were mean-centered and range-scaled (plot generated in Microsoft Excel 2016). To test the association between log-transformed metabolite concentrations and incidence of respiratory exacerbations, we built a multivariable logistic regression model for each metabolite adjusting for demographics (age and sex), study site, clinical factors that can affect metabolism (smoking status, BMI, diabetes mellitus, and current use of inhaled or oral corticosteroids)^42^, and clinical predictors of exacerbations (FEV_1_% predicted, history of respiratory exacerbations during the preceding year, white blood cell count, St. George’s Respiratory Questionnaire score and presence of gastroesophageal reflux disease)^25,38^. In addition to the multivariable logistic regression models above that dropped individuals with missing metabolite concentrations, we performed a multiple imputation sensitivity analysis that filled in missing metabolite concentration values (which comprised only 0.6% of all expected values) using the PROC MI function in SAS (Cary, NC, USA). The resulting 100 logistic regression outputs were combined using PROC MI ANALYZE which provided multiple imputation parameter estimates, confidence intervals and p-values. To test the association between log-transformed metabolite concentrations and each of FEV_1_% predicted, % emphysema on chest CT and 6MWD, we built multivariable linear regression models using the same covariates as above except for clinical predictors of exacerbations. Given that associations were tested for multiple metabolites, we corrected p-values for a false discovery rate (FDR) of 10% using the Benjamini-Hochberg method^43^. We used volcano plots to display the associations found in the multivariable models between metabolite concentrations and clinical parameters with effect size (beta coefficient for each metabolite) on the x-axis and −log_10_ of p-value on the y-axis (plots generated in R v3.4.0). We finally tested the association between a metabolite of interest (tryptophan) and each of the blood NLR, a known inflammatory biomarker in smokers^23^, and the BODE index, a well-validated prognostic tool in COPD^31^, after adjusting for age, sex, study site, smoking status, diabetes, inhaled or oral corticosteroid use, history of respiratory exacerbations during the preceding year, FEV_1_% predicted and BMI (the latter two variables were not included in the model for BODE given their inclusion in the outcome variable). All statistical analyses were performed in SAS v9.4 except when indicated.

### Ethical approval and informed consent

All methods were performed in accordance with the relevant guidelines and regulations. All participants gave written informed consent. The SPIROMICS protocol was approved by the institutional review boards of all participating institutions (Columbia University IRB 2, University of Iowa IRB-01, Johns Hopkins IRB-5, University of California Los Angeles Medical IRB 1, University of Michigan IRBMED B1 Board, National Jewish Health IRB, University of California San Francisco IRB Parnassus Panel, Temple University IRB A2, University of Alabama at Birmingham IRB #2, University of Illinois IRB #3, University of Utah IRB Panel Review Board 5, Wake Forest University IRB #5, University of North Carolina at Chapel Hill Non-Biomedical IRB).

## Supplementary information

Supplementary Material

## Data Availability

The datasets analyzed during the current study are available from the corresponding author on reasonable request.
