# Supplementary material for: Serum amino acid concentrations and clinical outcomes in smokers: SPIROMICS metabolomics study
Source: Sci Rep. 2019 Aug 6;9:11367. doi: 10.1038/s41598-019-47761-w (PMC6684630; doi:10.1038/s41598-019-47761-w)
Supplement: Supplementary file 1 — Supplementary Material [file 41598_2019_47761_MOESM1_ESM.docx]

**SUPPLEMENTARY MATERIAL**

**Serum amino acid concentrations and clinical outcomes in smokers:
SPIROMICS metabolomics study**

Wassim W. Labaki^1^*, Tian Gu^2^, Susan Murray^2^, Jeffrey L. Curtis^1,3^, Larisa Yeomans^4^,

Russell P. Bowler^5^, Graham Barr^6^, Alejandro P. Comellas^7^, Nadia N. Hansel^8^,

Christopher B. Cooper^9^, Igor Barjaktarevic^9^, Richard E. Kanner^10^, Robert Paine III^10^,

Merry-Lynn N. McDonald^11,12^, Jerry A. Krishnan^13^, Stephen P. Peters^14^, Prescott G. Woodruff^15^, Wanda K. O’Neal^16^, Wenqi Diao^17^, Bei He^17^, Fernando J. Martinez^18^, Theodore J. Standiford^1^, Kathleen A. Stringer^1,19#^, MeiLan K. Han^1#^

^1^ Division of Pulmonary and Critical Care Medicine, University of Michigan, Ann Arbor, MI, USA

^2^ Department of Biostatistics, University of Michigan, Ann Arbor, MI, USA

^3^ Medical Service, VA Ann Arbor Healthcare System, Ann Arbor, MI, USA

^4^ Biochemical NMR Core and the NMR Metabolomics Laboratory, College of Pharmacy, University of Michigan, Ann Arbor, MI, USA

^5^ Division of Pulmonary, Critical Care and Sleep Medicine, National Jewish Health, Denver, CO, USA

^6^ Division of Pulmonary, Allergy and Critical Care Medicine, Columbia University, New York, NY, USA

^7^ Division of Pulmonary, Critical Care and Occupational Medicine, University of Iowa, Iowa City, IA, USA

^8^ Division of Pulmonary and Critical Care Medicine, Johns Hopkins University, Baltimore, MD, USA

^9^ Department of Medicine, University of California Los Angeles, Los Angeles, CA, USA

^10^ Division of Pulmonary Medicine, University of Utah, Salt Lake City, UT, USA

^11^ Division of Pulmonary, Allergy and Critical Care Medicine, University of Alabama at Birmingham, Birmingham, AL, USA

^12^ Department of Genetics, University of Alabama at Birmingham, Birmingham, AL, USA

^13^ Division of Pulmonary, Critical Care, Sleep and Allergy, University of Illinois at Chicago, Chicago, IL, USA

^14^ Section on Pulmonary, Critical Care, Allergy and Immunologic Diseases, Wake Forest University, Winston-Salem, NC, USA

^15^ Division of Pulmonary, Critical Care, Allergy and Sleep Medicine, University of California San Francisco, San Francisco, CA, USA

^16^ Marsico Lung Institute, Department of Medicine, University of North Carolina at Chapel Hill, Chapel Hill, NC, USA

^17^ Department of Respiratory Medicine, Peking University Third Hospital, Beijing, China

^18^ Division of Pulmonary and Critical Care Medicine, Weill Cornell Medical College, New York, NY, USA

^19^ Department of Clinical Pharmacy, College of Pharmacy, University of Michigan, Ann Arbor, MI, USA

*** Corresponding author:**

Wassim W. Labaki

Division of Pulmonary and Critical Care Medicine

University of Michigan Health System

3916 Taubman Center

1500 E. Medical Center Drive

Ann Arbor, MI 48109

[wlabaki@med.umich.edu](mailto:wlabaki@med.umich.edu)

**^#^ Co-senior authors**

**Supplementary Table S1.** Mean log-transformed baseline concentrations (µM) of serum metabolites in the two exacerbation groups

| **Metabolites** | **KEGG**  **ID** | | **No exacerbation**  **(N = 109)** | | **≥ 1 exacerbation**  **(N = 29)** | | **p-value*** | | **FDR-corrected**  **p-value**** | |
| --- | --- | --- | --- | --- | --- | --- | --- | --- | --- | --- |
| O-acetylcarnitine | C02571 | 1.87 | | 1.60 | | 0.0002 | | 0.0054 | |  |
| Lysine | C00047 | 4.63 | | 4.42 | | 0.0006 | | 0.0054 | |  |
| 2-hydroxybutyrate | C05984 | 3.41 | | 3.03 | | 0.0006 | | 0.0054 | |  |
| Tryptophan | C00078 | 3.65 | | 3.42 | | 0.0011 | | 0.0074 | |  |
| Leucine | C00123 | 4.30 | | 4.06 | | 0.0032 | | 0.017 | |  |
| Ornithine | C00077 | 3.83 | | 3.64 | | 0.0054 | | 0.021 | |  |
| Valine | C00183 | 4.86 | | 4.67 | | 0.0060 | | 0.021 | |  |
| Choline | C00114 | 2.24 | | 2.02 | | 0.0061 | | 0.021 | |  |
| Serine | C00065 | 4.33 | | 4.13 | | 0.0089 | | 0.027 | |  |
| Glutamine | C00064 | 5.94 | | 5.80 | | 0.010 | | 0.027 | |  |
| Isoleucine | C00407 | 3.72 | | 3.56 | | 0.017 | | 0.042 | |  |
| Histidine | C00135 | 3.70 | | 3.56 | | 0.019 | | 0.043 | |  |
| Betaine | C00719 | 3.18 | | 2.96 | | 0.021 | | 0.044 | |  |
| Proline | C00148 | 4.96 | | 4.78 | | 0.028 | | 0.054 | |  |
| Phenylalanine | C00079 | 3.78 | | 3.65 | | 0.035 | | 0.062 | |  |
| Creatine | C00300 | 2.94 | | 2.70 | | 0.037 | | 0.062 | |  |
| Carnitine | C00318 | 2.74 | | 2.49 | | 0.050 | | 0.077 | |  |
| Threonine | C00188 | 4.28 | | 4.13 | | 0.051 | | 0.077 | |  |
| Tyrosine | C00082 | 3.66 | | 3.53 | | 0.063 | | 0.090 | |  |
| Glucose | C00031 | 8.03 | | 7.88 | | 0.068 | | 0.092 | |  |
| Creatinine | C00791 | 3.57 | | 3.43 | | 0.088 | | 0.11 | |  |
| Citrate | C00158 | 3.40 | | 3.30 | | 0.15 | | 0.18 | |  |
| Alanine | C00041 | 5.54 | | 5.44 | | 0.24 | | 0.28 | |  |
| Methionine | C00073 | 2.79 | | 2.73 | | 0.31 | | 0.35 | |  |
| Glycine | C00037 | 5.04 | | 5.00 | | 0.61 | | 0.66 | |  |
| Lactate | C00186 | 7.16 | | 7.12 | | 0.73 | | 0.76 | |  |
| Glutamate | C00025 | 3.88 | | 3.87 | | 0.90 | | 0.90 | |  |

* by two-sample t-test

** by the Benjamini-Hochberg method


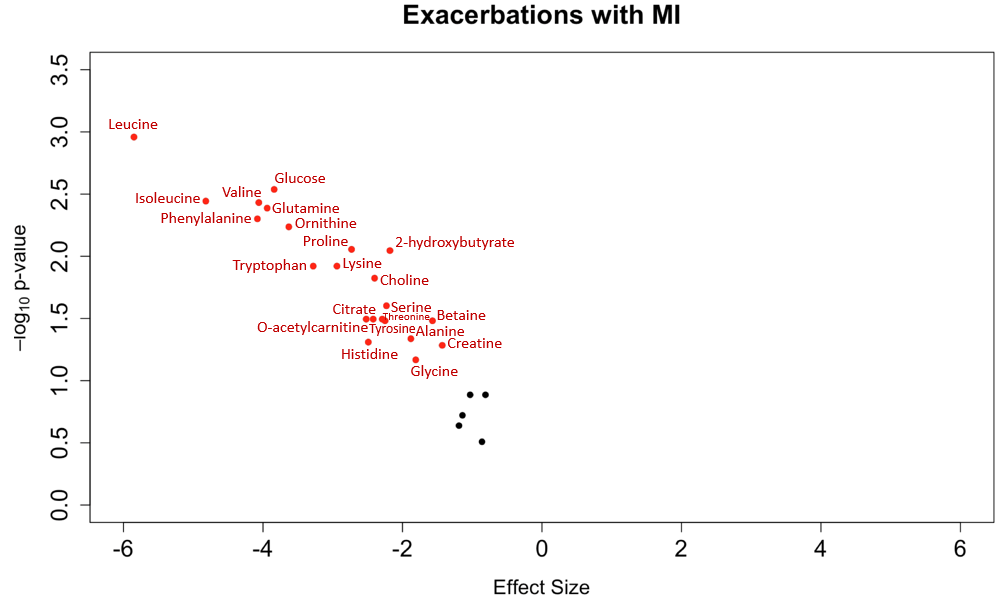


**Supplementary Figure S2.** Volcano plot showing the adjusted association between log-transformed metabolite concentrations and incident respiratory exacerbations from the multiple imputation (MI) sensitivity analysis. Red dots represent metabolites that are statistically significant at the 10% false discovery rate. Black dots represent metabolites that are not statistically significant.

224 blood samples selected for metabolomics analysis

31 samples excluded due to insufficient volume

193 samples assayed

24 samples excluded due to poor NMR spectra quality caused by:

- Significant volume loss during lyophilization (n=22)
- Other (n=2)

169 samples with adequate NMR spectra

12 samples excluded from this analysis due to never-smoking status of subjects

157 samples included in final analysis

**Supplementary Figure S3 – Consort Diagram**
